# A case report of successful treatment of 90° knee flexion contracture in a patient with adult-onset Still’s disease

**DOI:** 10.1186/s12893-016-0122-9

**Published:** 2016-02-09

**Authors:** Qiang He, Lin Xiao, Jianbing Ma, Guanghui Zhao

**Affiliations:** Department of Orthopaedic Surgery, Hong-Hui Hospital, Medical College of Xi’an Jiaotong University, Xi’an, China

**Keywords:** Total knee arthroplasty, Flexion contractures, Adult-onset Still’s disease, Serial casting, Case report

## Abstract

**Background:**

Severe knee flexion contractures greater than 80° are rare and challenging to manage. Previous studies have demonstrated unsatisfactory clinical results after correcting these deformities because residual flexion contractures were not corrected within a short period of time. We herein report the case of a patient with adult-onset Still’s disease with 90° of bilateral knee flexion contracture, which was successfully corrected by total knee arthroplasty and serial casting over a period of five weeks.

**Case presentation:**

A 47-year-old male was admitted to our orthopedic department for bilateral knee pain and a preoperative fixed flexion contracture of 90°. A diagnosis of adult-onset Still’s disease was made based on the patient’s medical history of a high spiking fever, salmon-colored rash and bilateral knee and wrist pain. Bilateral total knee arthroplasty was carried out to address these deformities, but residual flexion contracture was present. Subsequently, serial casting was used to achieve full extension at four weeks after surgery. Excellent function and patient satisfaction were observed at two years of follow-up.

**Conclusion:**

The new protocol of total knee arthroplasty with subsequent serial casting seems to be an efficient solution for knee flexion contractures greater than 80°. This report adds to the very small number of reported cases of adult-onset Still’s disease with severe knee flexion contractures and describes a patient who was successfully treated with a new protocol.

## Background

Adult-onset Still’s disease (AOSD), a rare systemic inflammatory disease, can cause joint destruction or flexion contractures [1, 2]. Most flexion deformities are mild and can be passively corrected at the time of surgery [3]. However, severe flexion contractures greater than 80°can been countered during total knee arthroplasty (TKA), although they are rare [4]. It is challenging to correct these contractures and bring the knees to full extension. Only a few cases of the management of severe contractures have been reported. These previous studies showed poor results because residual flexion contractures were not corrected within a short period of time [5, 6].

For several years, our institution has used serial casting to treat residual flexion contractures. Severe flexion contractures greater than 80° can be successfully corrected with a protocol involving TKA and serial casting. This protocol consists of the initial use of TKA, the application of serial casting, and knee mobilization.

In the present study, a new protocol was developed for the treatment of severe knee flexion contractures greater than 80°. The objective of this study was to investigate whether an AOSD patient who presented with 90° of bilateral knee flexion contracture can be successfully managed using this unique protocol.

## Case presentation

**Fig. 1:**
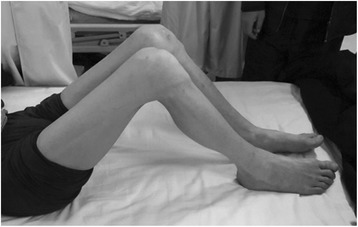
The preoperative condition of the lower limbs showing fixed flexion contracture of 90°

**Fig. 2:**
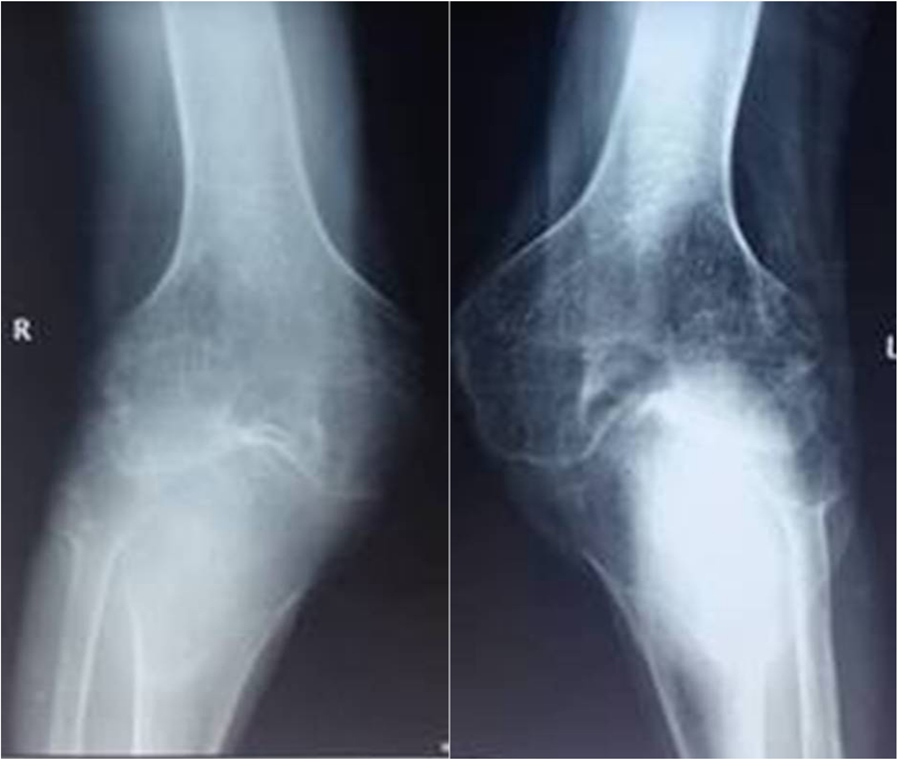
Preoperative anteroposterior radiographs of both knees showing complete complete disappearance of the joint spaces

A 47-year-old male was admitted to our orthopedic department for bilateral knee pain and preoperative fixed flexion contracture of 90°. He had been unable to walk for nearly 30 years. On examination, severe bilateral flexion deformity of the knee was observed (Fig. 1). The range of motion was −90° of extension and 135° of flexion in bilateral knees. The American Knee Society knee score (KSS) was 19. Plain radiography revealed almost complete disappearance of the knee joint space (Fig. 2). In addition, range of motion was normal in both ankles. The diagnosis of equines could be excluded.

The patient’s medical history revealed that he had been admitted to another hospital for a high spiking fever, salmon-colored rash and bilateral knee and wrist pain when he was 17 years old. The hematological studies showed leukocytosis (25,300 cells/mm^3^) and negative findings for rheumatoid factor. By the time he was 19 years old, the knee flexion contracture had progressed, and a fixed flexion contracture of 90° was present. We found that the patient’s past symptoms were consistent with Yamaguchi’s criteria for AOSD. The diagnosis of AOSD is based on clinical findings [7]. It requires compliance with Yamaguchi’s criteria, which have a sensitivity of 96.2 % and a specificity of 92.1 % for AOSD. The major criteria include a fever, arthralgia, rash and leukocytosis. The minor criteria include sore throat, lymphadenopathy, liver dysfunction and negative findings for rheumatoid factor [8]. After careful scrutiny of the patient’s medical history, we found that he satisfied four major and one minor diagnostic criteria based on the Yamaguchi criteria. He was therefore diagnosed with AOSD.

**Fig. 3:**
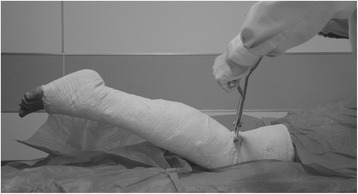
Postoperative photographs showing the serial casting from the thigh to ankle and the opening of the cast with plaster spreaders

**Fig. 4:**
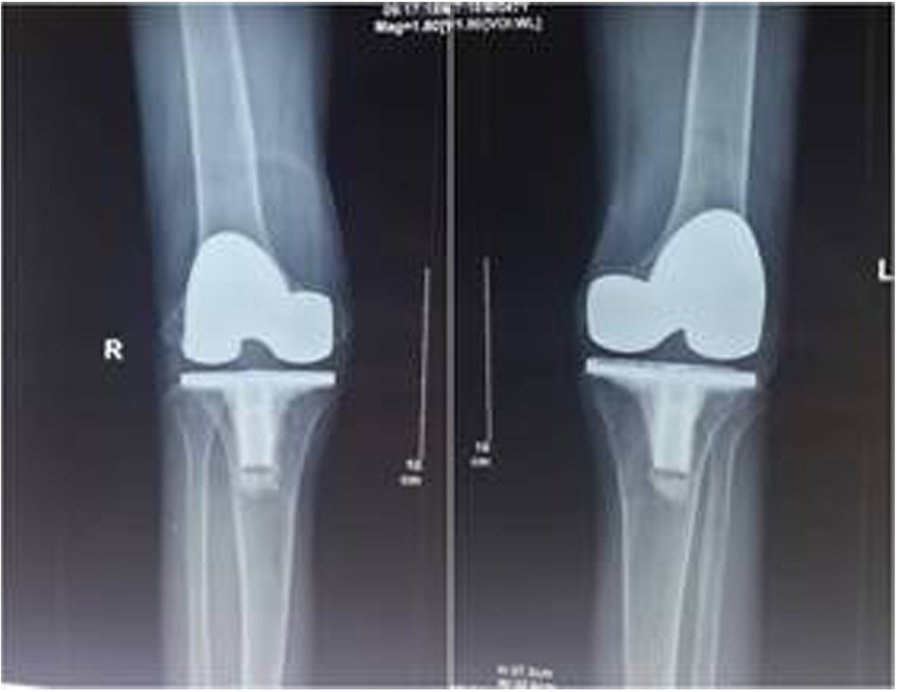
A postoperative anteroposterior radiograph showing satisfactory alignment of the prostheses

**Fig. 5:**
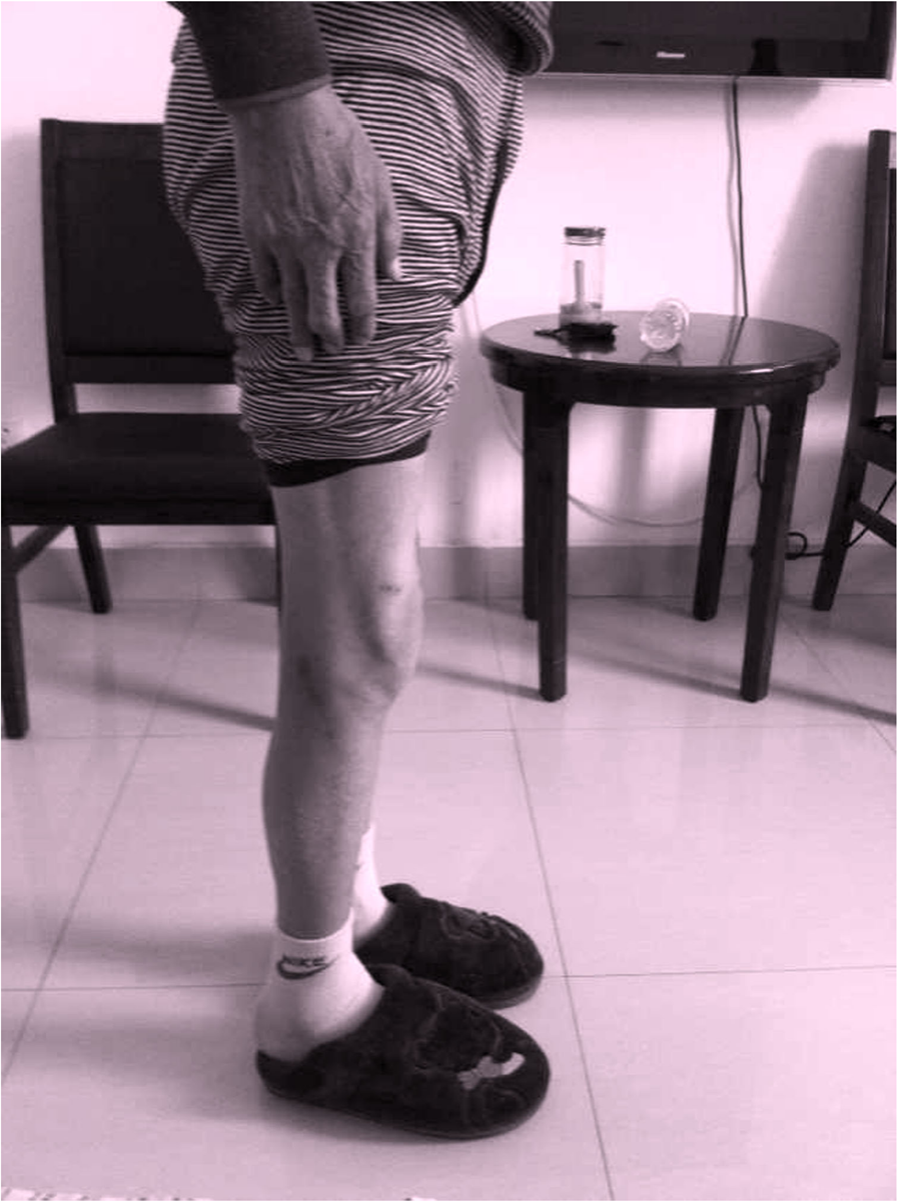
A photograph taken at the three-month follow-up exam demonstrating full extension of both knees

Our treatment protocol consisted of initial TKA, the application of serial casting, and knee mobilization. At first, bilateral arthroplasty was performed to avoid the difficulty of rehabilitating the first knee in the presence of a severe flexion contracture in the second knee. Both TKAs were implanted using midline incision and parapatellar approach, with posterior stabilized implants (NexGen, Zimmer, Warsaw, IN). To increase the extension gap, an additional 6 mm distal femoral resection and an additional 4 mm tibial resection were created. Removal of posterior osteophytes and posterior capsular release were performed after the bony cuts had been made. However, after performing these steps, it was noted that the knees still did not fully extend. Residual flexion contractures of 10° were present. Oral tramadol (37.5 mg) and paracetamol (325 mg) was given every eight hours for ten days postoperatively to reduce the patient’s pain and inflammatory reaction. Next, serial casting was used to achieve full extension for four weeks after surgery. The legs were immobilized in a long leg cast from the thigh to ankle. A posterior-based wedge was made at the level of the knee joint every two or three days, opening the cast by approximately 5° with plaster spreaders, and then completing the cast (Fig. 3). In this way, the residual flexion contractures were corrected gradually. This serial casting was done continuously during the first four weeks. Finally, knee mobilization using a continuous passive motion machine was performed twice a day for one week after the casts were removed. The range of motion of the bilateral knees had improved to 0° of extension and 110° of flexion at five weeks postoperatively. The KSS score for the bilateral knees was 70. No complications of the serial casting were noted. The radiographs revealed satisfactory alignment of the prostheses (Fig. 4). After discharge home, the patient received outpatient physical therapy services to prevent recurrence of the knee flexion contracture. At the three-month follow-up, the patient could ambulate without the use of assisting devices and was able to walk up and down stairs in a reciprocal manner. The range of motion was 0° of extension (Fig. 5) and 120° of flexion. The KSS score was 74. A recent two-year follow-up confirmed that the patient’s recovery was satisfactory and that the patient was still doing well in terms of the movement of both knees. The range of motion was 0° of extension and 120° of flexion. No recurrent flexion contracture was observed. The KSS score was 78.

## Discussion

The most important finding of the present study is a new protocol for successfully treatment of severe flexion contractures (greater than 80°). To our knowledge, this is the first report of adult-onset Still’s disease with 90° knee flexion contracture. Our findings also suggest that residual flexion contracture following TKA should be corrected within a short time to obtain satisfactory clinical outcomes.

In contrast to our present case, other studies have reported that treatment of these patients leads to poor results following various soft tissue treatments. In a recent study, the iliotibial tract and biceps tendon were elongated by a Z-plasty procedure before performing TKA in a patient with a flexion contracture of 85°. However, on the 20th postoperative day, the tibia dislocated posteriorly. This may have occurred because the extension angle was achieved within a very short period [6]. Another study indicated that severe flexion contracture greater than 90° could be treated with TKA, but that residual flexion contractures were observed postoperatively. The average residual flexion contracture was 7° after four years of follow-up [5]. Those studies demonstrated that TKA should be performed before Z-plasty to avoid the development of serious complications in these patients. It is important to achieve full extension within a short time as residual flexion contracture will persist and pose a functional disability [9].

TKA should be carefully performed in cases with 90° flexion contracture. All surgical techniques used during TKA to address preoperative flexion contracture were performed in the present case, including adequate bone resection, ligament releases, removal of posterior osteophytes, and posterior capsular releases [3]. It is common to perform an additional resection of the distal femur when treating severe flexion contracture. One study showed that for every 10° of flexion contracture, 2 mm of additional bone resection would be needed [10]. Therefore, a patient with an 80° flexion contracture would require 8 mm of distal bone resection. This is somewhat consistent with the current case, in which an additional 6 mm of bone was resected for the 90° flexion contracture. Some authors reported that a hinged implant may be necessary for preoperative flexion contractures greater than 30° [3]. Our patient did not need a hinged implant that would result in a greater amount of bone removed than a posterior stabilized implant. Second, serial casting was used after surgery to treat the residual flexion contracture for four weeks. Serial casting has been shown to result in significant improvements in joint contracture in patients with hemophiliac arthropathy [11]. In the current case, the goal of the serial casting after TKA was to achieve gradual and controlled extension as quickly as allowed, and then to maintain full extension for one to two weeks before removing the cast. These changes are thought to be the result of significant improvements in the tightening of the posterior capsule, the biceps femoris, the gatrocnemius muscles and tendons, as well as the collateral ligament [11]. Although applying serial casting was somewhat time-consuming, its cost was comparatively low. Thermal injury should also be avoided when applying serial casting. Moreover, the casting should be done continuously for no more than five weeks to avoid the complication of knee stiffness [12]. Various orthoses have been used in the past, including stretch splints to hold the joint in extension after TKA [13, 14]. However, these splints can cost more than $2000 and require prolonged treatment (e.g., four to55 weeks) [14]. In the current case, our patient achieved full extension in five weeks.

## Conclusions

The new protocol appears to be an efficient solution for knee flexion contractures greater than 80°. It led to excellent function and patient satisfaction at two-year follow-up. Severe knee flexion contractures can be corrected in a relatively short period of time without complications.

## Consent

Written informed consent was obtained from the patient for publication of this case report and any accompanying images. A copy of the written consent form is available for review by the editor of this journal.

## Ethics statement

The research protocol was approved by the Hong-Hui Hospital Human Research Ethics Committee (Permit Number: 15–269).

## Abbreviations

AOSD: adult-onset Still’s disease
KSS: The American Knee Society knee score
TKA: total knee arthroplasty
